# A New Role for Public Health in Bioterrorism Deterrence

**DOI:** 10.3389/fpubh.2014.00278

**Published:** 2014-12-10

**Authors:** Margaret E. Kosal

**Affiliations:** ^1^Georgia Institute of Technology, Sam Nunn School of International Affairs, Atlanta, GA, USA

**Keywords:** bioterrorism, biosecurity, bioweapons, national security, public health, deterrence, polio, Ebola

When this commentary was submitted in April 2014, only a handful of scholars and policy-makers in the defense and security communities were following the Ebola outbreak in West Africa, which was over 4 months old at that time. Now that thousands of people have died, cases have spread to the US and Europe, and thousands of US uniformed military are being deployed on humanitarian assistance/disaster relief missions, attention and interest are significantly heightened. The events of the last few months demonstrate the criticality for interdisciplinary thinking, which is more challenging due to different historical contexts, knowledge bases, interests, lexicon, and perspectives.

This commentary will explore the creation of new relationships between deterrence, infectious disease, and public health to reduce the threat of biological terrorism and increase international security. Examining the global spread of re-emerging infectious disease, such as the re-emergence of polio from northern Nigeria, offers a novel case study for thinking about how to deter potential bioterrorists who seek to use infectious disease. Polio outbreaks have more directly affected the developing world compared to the US or other nations with robust public health sectors. This example suggests that a bioterrorist attack would also be more devastating for developing countries in low-resource settings compared to the western world. Credibly, communicating this may offer a new approach to deterring bioterrorism by foreign actors. Although a robust public health sector has long been noted to reduce the vulnerability to a bioterrorism attack, actively promoting the strength of US public health can also serve as a powerful deterrent in its own right. The issue of terrorist groups utilizing biological weapons against other states is a mounting concern, yet little deterrence research in the field of political science addresses methods of dealing with the threat of bioterrorism. Thus, creating new conversations among the life sciences, public health, and political science can lead to new perspectives on deterring bioterrorism.

The issue of bioterrorism deterrence, if addressed, has been often added or subsumed under the auspices of deterrence strategies associated with nuclear weapons. In the second half of the twentieth century, nuclear deterrence dominated geopolitics and national security strategies. At its height, the threat of mutually assured destruction (MAD) existed in which both superpowers possessed arsenals with second-strike capabilities, i.e., the ability to respond to a first nuclear strike on land via use of nearly undetectable submarine-launched ballistic missiles with nuclear warheads.

These historical approaches, however, undermine and oversimplify the distinct challenges of deterring bioterrorism. One such method attempted is focusing on pathogen security, or securing and denying access to the materials necessary to develop biological weapons (i.e., deterrence by denial). Based on the nuclear non-proliferation model, pathogen security strives to control the materials, equipment, and personnel involved with production and use of biological agents. With nuclear weapons, controlling fissile materials proved successful because of key characteristics of the critical materials: fissile material is man-made and can be tracked. Those same characteristics that make nuclear weapons easier to track are those that make biological weapons material difficult to monitor. These characteristics include the presence of biological agents in nature, lower production costs, increased diversity of materials that could be used in bioweapons attacks, and multiple legitimate uses for biological materials. These differing features have not always been fully considered by policy-makers (1). Rather than focusing solely on securing biological materials and laboratories from misuse, other recommendations and strategies that the US has pursued include prevention measures such as biosurveillance, global laboratory and research cooperation, research and development of diagnostics and countermeasures, international stockpiles of effective medical countermeasures, and increased response and mitigation capabilities (2–6). These approaches aim to reduce consequences of an attack, afford earlier detection, and reduce vulnerability; they do not address the challenge of deterring use and reducing motivation directly, however.

To date, discussions about public health and deterrence have focused on measures such as regular vaccinations; access to timely medical care to treat infected, isolate suspected infected, and mitigate the spread of disease; confidence in the professional nature of health providers, etc. These are largely passive, defensive deterrence measures, in that they demonstrate credible capacity by a state to respond and mitigate the consequences of an attack (post-exposure) or reduce vulnerability to an attack by making it ineffective (pre-exposure) (7–9). Both approaches mentioned thus far, pathogen security and a defensive approach to terrorism, which ultimately aim to decrease vulnerability by fortifying civilian populations, are examples of deterrence by denial adapted from the realm of nuclear deterrence.

In contrast to these passive approaches, active deterrence strategies have not been explored. Active deterrence is actions and policies preventing a specific opponent from doing something they may wish to do. Traditionally, robust active deterrence has involved the application of expressive force to change the policy or character of the target government or group (10). Forces and policies are used to send a political message. In contrast to passive strategies, active deterrence is more dynamic and may incorporate escalating threats in response to an adversary. What this would look like at the nexus of international security and public health is largely an unexplored area of study or policy. Therefore, there are limited models for thinking about deterrence that have been developed exclusively for bioterrorism. As a consequence, the role of a robust public health system for twenty-first century active deterrence remains to be explored. There has not been a substantive consideration of robust public health system as a strategic asset in a more active deterrence role.

The threat of inflicting punishing retaliation against some aggressor, not the ability to prevent some hostile act from occurring, is the core of traditional deterrence theory. Within new deterrence approaches in political science, however, there are several types of definable strategies that may be applied to bioterrorism by foreign actors (11). *Indirect deterrence* focuses on third party players and their roles in terrorist attacks. Third parties are most typically state sponsors or supporting financiers. This concept is based on the recognition that while a terrorist may be willing to die for his cause, it is less likely that explicit and tacit supporters are willing to pay a similar retribution. Appealing to or directing bioterrorism deterrence efforts toward tacit supporters is an untapped area. *Collective actor deterrence* utilizes the power and influence of institutions like the United Nations, NATO, or other broad coalitions to deter terrorist actions, highlighting the legitimacy of the organization and the international community rather than the interests of a single state. For bioterrorism, the WHO and African Union’s disease eradication efforts are examples. *Internalized deterrence* plays off the psyche of a terrorist, combining abstract concepts of criminology and social constructivism to subconsciously deter a terrorist through social taboos and norms (12, 13). This might involve leveraging fear of disease spreading to oneself or one’s own community. *Tailored deterrence* attempts to individualize each situation to reach the best possible solution, leveraging cultural, political, social, and other specific knowledge. These newer deterrence strategies offer opportunities for dealing with bioterrorism threats by foreign actors, which could be combined with public health information and resources.

In thinking about public health infrastructure as an active or passive part of new deterrence strategies, it is useful to think about the role of missile defense. As the presence of a ballistic missile defense system is supposed to be an existential deterrent itself, so could be a strong public health system. Missile defense is both a passive deterrent and, if used, an active deterrent, as it stops something from occurring. A strong public health infrastructure is likely to be the key in reducing the vulnerability to bioterrorism attack, as well as having a potential role in deterring a foreign terrorist group from even considering such an attack. If a biological weapon launched by a terrorist group will have little or no effect on the target country because of a known robust public health sector, then a foreign terrorist may be discouraged from launching a biological weapons attack in the first place. If foreign terrorists are also aware of the weak public health infrastructure with their own borders, and the increased risks to them and their publics in the event of an accident in developing biological weapons and/or spread of an infectious disease that they might launch, this may also deter them from pursuing this work. In addition, even the accidental release of a dangerous pathogen or the spread of an infectious disease via attack will most likely cause disproportional negative effects to nations with limited public health infrastructures and affect tacit and explicit supporters in those states.

The role of a robust public healthcare system for its deterrence capacity can be explored through empirically driven case study methods against predominant theories of deterrence in political science (14, 15) and in comparison to other works considering the possibility of deterring bioterrorism (16–20). For example, the re-emergence of polio offers a potentially useful example to think about the effects of a potential bioterrorist attack on the developed and the developing world. Polio is both a contagious infectious disease and transmissible from human-to-human (like smallpox and plague). The poliovirus is highly transmissible with a basic reproductive rate or secondary transmission rate (R0) exceeding most suspected biological agents, e.g., standard estimates of R0 for polio range from 5 to 7 (21, 22), whereas R0 for suspected bioterrorist agents like smallpox (1.8–3.2) (23–25); pneumonic plague (0.8–3.0) (26, 27); and even Ebola (1.34–2.0) (28, 29) are lower. It is not a likely biological terrorism agent, however, due to the low-mortality associated with infection. It is, however, a useful model for thinking about the spread of infectious disease and the importance of a robust public health infrastructure as a deterrence strategy.

At the beginning of 2003, the complete eradication of polio appeared to be within the grasp of the World Health Association and its many partners. In 1998, the World Health Organization estimated there were over 365,000 new cases of polio; by early 2003, the rate of infection had declined to <1,000 new cases worldwide due to a vigilant vaccination effort (30). That trend was interrupted, however, when Nigerian citizens refused to be vaccinated after hearing unfounded allegations of contaminated vaccines that would lead to sterility or cause HIV/AIDs. Before 2003, polio had largely been confined to only a handful of countries; Nigeria, India, Pakistan, and Afghanistan accounted for 93% of the world’s cases (31). What started with the refusal of local clerics to allow vaccination led to the reestablishment or importation of the poliovirus to 14 countries that were previously disease-free.

Transport of the contagious virus was not limited to neighboring African states. The poliovirus moved through Sudan to Ethiopia crossing the Red Sea to Lebanon and Yemen. The latter was been particularly severely affected, witnessing more than 500 new cases in the first half of 2005. The poliovirus spread as far as Indonesia, where it afflicted more than 150 people in a single year in 2 provinces, predominantly children (32). Prior to this outbreak, Indonesia had been polio free for nine years. Genetic fingerprinting confirmed that the strain imported to Indonesia came from northern Nigeria through Sudan, most closely resembling an isolate recovered in Saudi Arabia in December 2004. A pilgrim returning from Mecca or a returning foreign worker is suspected to have brought the virus to the island of Java, across an ocean and thousands of miles from its source. The polio virus continues to persist in a limited number of states in the developing world, specifically in Nigeria, Afghanistan, and Pakistan, where a ban on vaccination by Islamist leaders in Waziristan remains in place. Since 2013, polio (linked genetically to the strain in Pakistan) has spread from Syria to Iraq (33).

Countries that have witnessed the re-emergence of poliovirus outbreaks have some crucial links: social and political challenges that have impeded the development and implementation of appropriate public health infrastructures and measures. Not unexpectedly, there is an inverse relationship between government health expenditure in health and number of polio cases.

Looking at the spread of polio can provide us with a lens to think about the impacts of bioterrorism in states with developed public health infrastructures and those who do not. A bioterrorist attack, especially one with a contagious agent like smallpox or pneumonic plague, will likely impact the developing parts of the world substantially more than the US. One only has to look as far as polio’s re-emergence (or more recently the outbreak of Ebola virus disease in West Africa) to see the very real repercussions of a contagious virus and how the most dire causes and effects of infection and spread stem from poor public health infrastructures (34).

Creating a new deterrence strategy for bioterrorism is needed. Credibly, communicating the differential capacities to respond and the comparative likely outcomes will require diplomacy, coordination with civil affairs, specialized knowledge of individual states, and regions of the developing world. These are fundamentally interdisciplinary efforts that should leverage small teams from diplomatic, development, public health, and defense communities. One single parochial voice will be inadequate. Further improving the US domestic public health infrastructure would be beneficial and cost effective regardless of whether an outbreak is intentional or natural. The devastating Ebola outbreaks serve as a call for urgent investment in public health infrastructures worldwide, to provide both responsive and proactive actions to deter bioterrorism and to deal with natural disease outbreaks. Public health remains a powerful and often underutilized asset for bioweapons defense through vulnerability reduction; leveraging public health may also enable new approaches to deterring bioterrorism threats. International security scholars would benefit from better understanding of and leveraging the knowledge of the public health community.

## Conflict of Interest Statement

The author declares that the research was conducted in the absence of any commercial or financial relationships that could be construed as a potential conflict of interest.

## References

[1] Ostfield M Pathogen security: the illusion of security in foreign policy and biodefense. Int J Risk Assess Manag (2009) 12:205–10 10.1504/IJRAM.2009.025919

[2] National Strategy for Countering Biological Threats. Washington, DC (2009). Available from: www.whitehouse.gov/sites/default/files/National_Strategy_for_Countering_BioThreats.pdf

[3] National Strategy for Biosurveillance. Washington, DC (2012). Available from: http://www.whitehouse.gov/sites/default/files/National_Strategy_for_Biosurveillance_July_2012.pdf

[4] United States Government Policy for Oversight of Life Sciences Dual Use Research of Concern (2012). Available from: http://www.phe.gov/s3/dualuse/Documents/us-policy-durc-032812.pdf

[5] Public Health Security and Bioterrorism Preparedness and Response Act of 2002, Public Law 107-188 (2002). Available from: http://www.gpo.gov/fdsys/pkg/PLAW-107publ188/pdf/PLAW-107publ188.pdf

[6] Department of Defense Strategy for Countering Weapons of Mass Destruction. Washington, DC (2014). Available from: http://www.defense.gov/pubs/DoD_Strategy_for_Countering_Weapons_of_Mass_Destruction_dated_June_2014.pdf

[7] Atlas RM Combating the threat of biowarfare and bioterrorism: defending against biological weapons is critical to global security. Bioscience (1999) 49:465–77 10.2307/1313554

[8] Hamburg MA. Bioterrorism: a challenge to public health and medicine. J Public Health Manag Pract (2000) 6:38–44. 10.1097/00124784-200006040-00007 10977611

[9] Khan AS Morse S Lillibridge S Public-health preparedness for biological terrorism in the USA. Lancet (2000) 356:1179–82 10.1016/S0140-6736(00)02769-0 11030310

[10] Schelling TC Arms and Influence. New Haven, CO: Yale University Press (1966).

[11] Knopf JW The fourth wave in deterrence research. Contemp Secur Pol (2010) 31:1–33 10.1080/13523261003640819

[12] Kosal ME Finnel S The role of ethical discourse in the contested common of radical Islam & WMD terrorism. Paper Presented at the Annual Meeting of the International Studies Association Annual Conference Global Governance Montreal, QC: Political Authority in Transition (2011).

[13] Hashmi SH Lee SP Ethics and Weapons of Mass Destruction. Cambridge, UK: Cambridge University Press (2004). 552 p.

[14] Trager RF Zagorcheva DP Deterring terrorism: it can be done. Int Secur (2006) 30:87–123 10.1162/isec.2005.30.3.87

[15] Levine DK Levine RA Deterrence in the cold war and the ‘War on Terror’. Defence Peace Econ (2006) 17:605–17 10.1080/10242690601025526

[16] Davis K Jenkins BM Deterrence and Influence in Counterterrorism: A Component in the War on al Qaeda. Santa Monica, CA: RAND (2002). 109 p.

[17] Joseph RG Reichart JF Deterrence and Defense in a Nuclear, Biological, and Chemical Environment. Washington, DC: National Defense University Press (1999). 44 p.

[18] Koblentz G Pathogens as weapons: the international security implications of biological warfare. Int Secur (2003) 28:84–122 10.1162/016228803773100084

[19] Galamas F Biological weapons, nuclear weapons and deterrence: the biotechnology revolution. Comp Strat (2008) 27:315–23 10.1080/01495930802358364

[20] Martin S The role of biological weapons in international politics. J Strat Stud (2002) 25:63–98 10.1080/714004040

[21] Fine PE Herd immunity: history, theory, practice. Epidemiol Rev (1993) 15:265–302. 8174658 10.1093/oxfordjournals.epirev.a036121

[22] Eisenberg JNS Brookhart MA Rice G Brown M Colford JM Jr. Disease transmission models for public health decision making: analysis of epidemic and endemic conditions caused by waterborne pathogens. Environ Health Perspect (2002) 110:783–90. 10.1289/ehp.02110783 12153759 PMC1240949

[23] Halloran ME Longini IM Jr Nizam A Yang Y Containing bioterrorist smallpox. Science (2002) 298:1428–32 10.1126/science.1074674 12434061

[24] Bozzette SA Boer R Bhatnagar V Brower JL Keeler EB Morton SC A model for a smallpox-vaccination policy. N Engl J Med (2003) 348:416–25. 10.1056/NEJMsa025075 12496353

[25] Meltzer MI Damon I LeDuc JW Millar JD. Modeling potential responses to smallpox as a bioterrorist weapon, appendix I: a mathematical review of the transmission of smallpox. Emerg Infect Dis (2001) 7:i–vi. 10.3201/eid0706.010607 11747722 PMC2631899

[26] Gani R Leach S. Epidemiological determinants for modeling pneumonic plague outbreaks. Emerg Infect Dis (2004) 10:608–14. 10.3201/eid1004.030509 15200849 PMC3323083

[27] Kool JL. Risk of person-to-person transmission of pneumonic plague. Clin Infect Dis (2005) 40:1166–72. 10.1086/428617 15791518

[28] Chowell G Hengartner NW Castillo-Chavez C Fenimore PW Hyman JM. The basic reproductive number of Ebola and the effects of public health measures: the cases of Congo and Uganda. J Theor Biol (2004) 229:119–26. 10.1016/j.jtbi.2004.03.006 15178190

[29] Nishiura H Chowell G Early transmission dynamics of Ebola virus disease (EVD), West Africa, March to August 2014. Euro Surveill (2014) 19(36):20894 10.2807/1560-7917.es2014.19.36.20894 25232919

[30] Samba E Nkrumah F Leke R Getting polio eradication back on track in Nigeria. N Engl J Med (2004) 350:645–6 10.1056/NEJMp038210 14960740

[31] Minor PD Polio eradication, cessation of vaccination and re-emergence of disease. Nat Rev Microbiol (2004) 2:473–82 10.1038/nrmicro906 15152203

[32] Pallansch MA Sandhu HS The eradication of polio – progress and challenges. N Engl J Med (2006) 355:2508–11 10.1056/NEJMp068200 17167133

[33] Arie S Polio virus spreads from Syria to Iraq. BMJ (2014) 348:g2481 10.1136/bmj.g2481 24696176

[34] Boozary AS Farmer PE Jha AK The Ebola outbreak, fragile health systems, and quality as a cure. JAMA (2014) 312:1859–60 10.1001/jama.2014.14387 25285459

